# Facilitators and obstacles in instituting of early identification and brief intervention in Catalonia

**DOI:** 10.1186/1940-0640-7-S1-A33

**Published:** 2012-10-09

**Authors:** Lídia Segura, Estela Diaz, Jorge Palacio, Rosa Freixedas, Nuria Bastida, Eulalia Duran, Antoni Gual, Joan Colom

**Affiliations:** 1 Program on Substance Abuse, Catalonia Department of Health, Barcelona, Spain; 2 Working Group on Alcohol, Catalan Society of Family and Community Medicine, Catalonia Department of Health, Barcelona, Spain; 3 Hospital Clínic of Barcelona Addictions Unit, Barcelona, Spain

## 

In Catalonia, the implementation of early identification and brief intervention (EIBI) started in 2002. Since then, through an iterative process consisted of two consecutive phases, significant improvements have been achieved in the number of professionals involved, in the level of screening conducted in primary care, and in the referral of people with alcohol use disorders to specialty care. Nevertheless, some elements still need to be addressed to achieve widespread adoption of EIBI. In this presentation, we review the elements identified as facilitators and obstacles to this process. The information comes from process indicators, from ongoing evaluation of results following implementation of the program, and from qualitative information gathered through surveys and interviews with participants in the referral network. Among the facilitators are the creation of the alcohol treatment referral network and the ongoing support for this network, peer-to-peer training, coordination between primary and specialist care, and inclusion in contractual incentives. Barriers included diversity of medical records available, lack of standardized tools for monitoring of action, and lack of a reliable system to monitor implementation in primary care. Once adequate training is available and a positive attitude toward EIBI has been achieved for all involved, one of the chief obstacles facing any health-care system is the institutionalizing the necessary work tools in the available clinical histories. In Catalonia this represents a challenge since there are 27 providers using nine different MR, with the resulting difficulties for integrating and monitoring results.

